# Neural Dynamics of Cognitive Control in Various Types of Incongruence

**DOI:** 10.3389/fnhum.2020.00214

**Published:** 2020-06-05

**Authors:** Liufang Xie, Bihua Cao, Zixia Li, Fuhong Li

**Affiliations:** School of Psychology, JiangXi Normal University, Nanchang, China

**Keywords:** cognitive control, flanker task, rule switching, response switching, P300, N2

## Abstract

Conflict-control is a core function of cognitive control. Although numerous studies have considered cognitive control to be domain-general, the shared and distinct brain responses to different types of incongruence or conflict remain unclear. Using a hybrid flanker task, the present study explored the temporal dynamics of brain activation to three types of incongruence: flanker interference, rule-based response switch (rule-switch), and action-based response switch (response-alternation). The results showed that: (1) all three types of incongruence evoked larger N2 amplitudes than the congruent condition in the frontal region, with the N2 amplitudes and topographical distribution of the N2 effect differing between the different types of incongruence; and (2) in the P300 time window, the flanker interference condition yielded the most delayed P300 latency, whereas the rule-switch and response-alternation conditions yielded smaller P300 amplitudes with a longer interval from P300 peak to a keypress. These findings suggest that different types of incongruence are first monitored similarly by the cognitive control system and then resolved differently.

## Introduction

A hallmark of human goal-pursuit is the ability to stay focused on task-goals in the presence of incongruence or conflict information (Botvinick et al., 2001, 2004). Resolving these conflict situations requires one to not only detect the conflicts but also to make rapid decisions regarding how to react according to the current goals (Kerns et al., 2004; Larson et al., 2012; Dignath et al., 2015; Rey-Mermet et al., 2019). In recent decades, many researchers have shown great interest in conflict monitoring and resolution processes (Kerns et al., 2004; Croxson et al., 2009; Clayson and Larson, 2011; Dignath et al., 2015; Ho et al., 2019; Rey-Mermet et al., 2019).

One of the influential models, the conflict-monitoring (CM) model of cognitive control, describes a single, “all-purpose” conflict control loop consisting of a conflict monitor module and an executive control module (Botvinick et al., 2001; Rey-Mermet et al., 2019; Schuch et al., 2019). According to the CM model, various types of conflicts will yield highly similar patterns of brain activation because they share a centralized module of cognitive control. The CM model is supported by the image studies that have demonstrated the pivotal role of the medial prefrontal cortex (mPFC) in implementing and monitoring higher-order cognitive processes (Shenhav et al., 2013; Silvetti et al., 2014, 2018; Vassena et al., 2014). Ongoing research efforts are focused on the construction of a new conceptualization of mPFC to provide a comprehensive account of its functions including conflict monitoring and cognitive control, value valuation, reward prediction, reinforcement learning, and emotional regulation (Silvetti et al., 2014, 2018). The theoretical (*via* computational modeling) and empirical evidence supporting the role of the mPFC as an action-outcome comparator for the past 15 years have been summarized (for a review, Silvetti et al., 2014). Moreover, recent findings encompass certain elements of different domains including prediction errors, outcome coding, and effort optimizer (cost-benefit valuation), which are cardinal components of all goal-directed behavior, to formulate a unifying comprehensive account of the existing literature (Croxson et al., 2009; Shenhav et al., 2013; Vassena et al., 2014; Silvetti et al., 2018). Similarly, subregions of mPFC, such as the dorsal-anterior cingulate cortex (dACC), which has been implicated in a diversity of functions, from performance monitoring to the execution of control and action selection, was also implicated in the diverse array of findings under a single modal. Shenhav et al. (2013) suggested that the diverse functions of dACC can be understood in terms of a single underlying function: allocation of control based on an evaluation of the expected value of control (EVC). In summary, recent research on the specific function (e.g., CM and resolution) of mPFC confirms the existence of a single underlying basis and the specific resource allocation of different tasks.

The current study will further explore how conflict monitoring and resolution can be adapted to specific task demands when dealing with different conflicts or types of incongruence (Lavie et al., 2004; Hübner et al., 2010; Rey-Mermet et al., 2019). Specifically, we tried to compare the cognitive processes of three different incongruent information in a rule-shifting flanker task to observe how the CM system evaluates different conflict information and invests cognitive resources to resolve conflicts.

Typically, the flanker task, task switching, and the hybrid paradigms have been extensively used to explore the various subprocesses of cognitive control, such as performance monitoring, behavior adaptation, conflict control, attentional switching, and task-set reconfiguration (TSR; Morimoto et al., 2008; Dignath et al., 2015; Von der Gablentz et al., 2015; Freund and Nozari, 2018; Richardson et al., 2018; Rietbergen et al., 2018; Ho et al., 2019; Rey-Mermet et al., 2019), which involve several different types of incongruence processes. For example, in a hybrid rule-shifting flanker task, the response rule (e.g., left-hand response to “↑” and right-hand response to “↓”) would reverse after several rule-repeat blocks or trials (Rushworth et al., 2002a; Umebayashi and Okita, 2010; Schroder et al., 2012; Von der Gablentz et al., 2015; Xie et al., 2017; Richardson et al., 2018; Ludyga et al., 2019). In this task, not only should the flanker interference, the stimulus-response (S-R) conflict between the target and flankers be resolved (Eriksen, 1995), but cognitive control of the following two types of response-related incongruence processes is also required: the response-alternation (Swainson et al., 2006) and the rule-switch (Rushworth et al., 2002a). The purpose of the present study is to elucidate the shared and distinct brain activation patterns of cognitive control in these three types of incongruence processes.

In the canonical flanker task, which involves perceptual conflicts and response competition, there may be at least two primary cognitive processes, that is, stimulus evaluation and response processes including response selection and execution (Coles et al., 1985; Gratton et al., 1988; Smid et al., 1990; Stürmer et al., 2002; Rey-Mermet et al., 2019; Schuch et al., 2019). During stimulus evaluation, both the target and context (or distractor) information is processed in the cognitive system. When the two different parts of the information are incongruent (e.g., flankers and the target are dissimilar), this will activate the neural network of conflict monitoring in the frontal cortex and evoke an enhanced N2 component (Kopp et al., 1996; Heil et al., 2000; Botvinick et al., 2001, 2004; Kerns et al., 2004; Bartholow et al., 2005; Clayson and Larson, 2011). As compared to congruent trials, stimulus evaluation is harder in incongruent trials, which was reflected in the longer P300 latency (Coles et al., 1985; Donchin and Coles, 1988; Smid et al., 1990; Ridderinkhof and van der Molen, 1995; Kopp et al., 1996; Heil et al., 2000; Van’t Ent, 2002; Umebayashi and Okita, 2010; Mückschel et al., 2017). Accordingly, some investigators have suggested that P300 latency is more sensitive to stimulus evaluation than to response selection and execution (McCarthy and Donchin, 1981; Magliero et al., 1984; Coles et al., 1985).

In addition to incongruence from the flankers, other conflict information such as response alternation (e.g., the hand response in the current trial is different from that of the preceding trial) can be monitored by the conflict monitoring system (Swainson et al., 2006). Previous studies have found that identical responses typically evoked particularly fast responses, reflecting the remission of the same response (Bertelson, 1963; Pashler and Baylis, 1991; Hommel and Colzato, 2004). Moreover, previous studies on flanker interference have seldom considered whether the response in the current trial is switched or repeated when compared with the preceding trial. A response switch, which is referred to as a response-alternation, will be treated as an independent incongruent piece of information in the current study. Previous studies found that when the stimulus and response are different from that of the preceding trial, the response alternation triggered the CM system, resulting in a slow response (Bertelson, 1963; Meiran, 2000; Xie et al., 2017) and a slightly larger N2 amplitude (Gajewski et al., 2010). In an arrow-direction discrimination task, Swainson et al. (2006) found a reduced P300 for a response alteration as compared that of response repetition in a fixed two-trials alternation runs the task-switching experiment. Additionally, in a repetition-priming task of word-classification, Race et al. (2010) found that the S-R repetition effect was independent of the stimulus repetition effect and the stimulus and response change (novel) condition elicited more negative amplitudes than the S-R repetition condition within the 450–500 ms interval after stimulus onset.

Another type of response-related incongruence in a hybrid rule-shifting flanker task is referred to as rule-switch, which is a form of set-shifting that involves switching or reversing the response rule (Rushworth et al., 2002a; Crone et al., 2005; Parris et al., 2007; Von der Gablentz et al., 2015; Xie et al., 2017; Shi et al., 2018). For example, if the S-R rule was “S1-R1, S2-R2” before the rule-switch trial, participants responded to S1 with R1. Then, in the rule-switch trial, the rule is reversed (e.g., S1-R2 and S2-R1), and participants would respond to S2 with R1. The processing of rule-switch is related to several event-related potential (ERP) components including P2, N2, and P300. P2 is sensitive to an early task-set updating process that would rapidly “detect” a relevant change in the task when a shift is involved (Capizzi et al., 2015; Tsai and Wang, 2015). The enhanced N2 component in the frontal cortex reflects the detection of conflict in the task or rule (Gajewski et al., 2010; Schroder et al., 2012; Kieffaber et al., 2013; Richardson et al., 2018). The P300 component is suggested to be related to the rehearsal and implementation of task rules in working memory (Barceló et al., 2002).

To summarize, the current study adopted a rule-shifting flanker task to explore the neural distinction underlying the cognitive processes of incongruent information in flanker interference, rule-switch, and response-alternation conditions. Based on the aforementioned studies, the three types of incongruence seemed to be commonly associated with two ERP components, that is, N2 and P300. Thus, the following predictions were made. First, various types of incongruence share a centralized module of cognitive control, so they yield highly similar patterns of brain activation (Botvinick et al., 2001), which were expected to be observed in the N2 time window. That is, all three types of incongruence might evoke an enhanced N2 component that is related to conflict monitoring (Bartholow et al., 2005; Gajewski et al., 2010; Richardson et al., 2018). Second, after being monitored by the cognitive control system, the three types of incongruence are assumed to be resolved in the late time window (P300 component). Since conflict resolution is adapted to specific task demands (Lavie et al., 2004; Hübner et al., 2010; Rey-Mermet et al., 2019), the brain reactions are expected to differ markedly in the P300 time window. Specifically, flanker incongruence is an S-S conflict, so it might be primarily resolved in the stage of stimulus evaluation (Kornblum et al., 1990; Kornblum, 1994). In contrast, conflicts in the rule-switch and response-alternation conditions are mainly related to response processes, so they might be completely resolved in the stage of response selection and execution. Therefore, we expected that the P300 latency that is closely associated with stimulus evaluation might be longer for the flanker interference trials as compared to the rule-switch and response-alternation trials (McCarthy and Donchin, 1981; Magliero et al., 1984; Coles et al., 1985). In contrast, for the rule-switch and response-alternation trials, the increased time in resolving the incongruence might primarily come from the stage after the stimulus evaluation (i.e., P300 latency). Third, the difference in reconfiguration between the rule-switch and response-alternation conditions might be additionally associated with the P300 or LPC amplitude difference (Swainson et al., 2006; Barceló et al., 2002; Race et al., 2010). Specifically, the reconfiguration in the rule-switch was about the task-set, which was more complicated than the single response selection in the response-alternation. In other words, the conflict in task rule is at a higher level than the conflict in the response (Kleinsorge and Heuer, 1999; Monsell and Driver, 2000; Elchlepp et al., 2013). We, therefore, expected that the incongruence in the rule-switch condition may be more difficult to resolve than that in the response-alternation condition in the late time window (Barceló et al., 2002; Swainson et al., 2006; Periáñez and Barceló, 2009; Race et al., 2010).

## Materials and Methods

### Participants

After obtaining informed written consent, 25 undergraduate volunteers participated (12 male subjects, aged 18–24 years, mean = 20.04 years, standard deviation = 1.34 years) in this study. All subjects reported being right-handed, and all had normal or corrected-to-normal eyesight and normal color vision. No subject reported neurological disorders. All participants were paid for their participation. The study was carried out following the recommendations of the Moral and Ethics Committee of the School of Psychology at Jiangxi Normal University (China) and the latest version of the Declaration of Helsinki.

### Materials, Experimental Design, and Procedure

**Figure 1:**
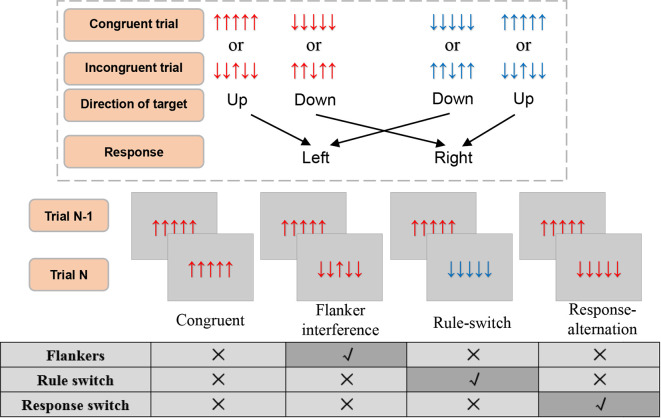
The illustration of experimental stimuli, condition, and response rule. The response rules switch every 6–10 trials. Responses should be elicited within the 1,000 ms during which the stimulus was presented. All four conditions had a congruent trial as trial N-1. Trial N can then be defined as a specific condition according to none or the single incongruence in it as compared with trial N-1. The other combinations of trial N-1 and trial N were all filling trials.

In a modified Eriksen flanker task (Eriksen and Eriksen, 1974; Xie et al., 2017), five vertical arrow strings consisting of “↑” and “↓” were used as congruent (↑↑↑↑↑, ↓↓↓↓↓) and incongruent stimuli (↓↓↑↓↓, ↑↑↓↑↑). In each trial, participants were instructed to focus only on the centrally presented arrow (i.e., to ignore the flankers) and respond by pressing either the “F” or the “J” button on the QWERTY keyboard. Participants were informed about the fixed S-R rule *via* the color of the stimulus, which was either red or blue. For example, if the stimulus is red, the response rule is pressing “F” for “↑” and pressing “J” for “↓”; if the stimulus is blue, the response rule is reversed. The color corresponding to the rules was counterbalanced. There was no feedback after the response. To prevent subjects from anticipating the response rule switch, the rule regarding the S-R associations switched every 6–10 trials. Subjects would use the new response rule indexed by the color of the stimulus (Figure 1). Participants were instructed to respond as quickly and accurately as possible.

For the present study, we adopted a single-factor experimental design. Based on the continuous stimulus sequence and the relationship with trial N-1 and trial N, the following four conditions (see Figure 1) were defined : (1) Flanker interference, in which flanker incongruence was processed during the presentation of the current trial, where there was no other incongruence, such as rule or response switch. In this condition, the current trial was an incongruent stimulus (e.g., ↓↓↑↓↓) preceded by a congruent stimulus (e.g., ↑↑↑↑↑) that had the same S-R rule and action as the current trial; (2) Response-alternation, in which the incongruence came from response switch according to the preceding trial, and where there was no flankers incongruence or rule switch. In this condition, the current trial was a congruent stimulus (e.g., ↑↑↑↑↑) preceded by a congruent stimulus (e.g., ↓↓↓↓↓) with the same S-R rule but a different action; (3) Rule-switch, in which the rule switch was processed, and there was no flankers or response switch. In this condition, the current trial was a congruent stimulus preceded by a congruent stimulus that had the same response but a reverse S-R rule; and (4) Congruent condition, in which no incongruence was processed. In this condition, the current trial and the preceding trial were both congruent stimuli with the same response and the same S-R rule. Taken together, the definition of each condition was based on the same stimuli in the preceding trial (i.e., a congruent trial of “↑↑↑↑↑” or “↓↓↓↓↓”), which ensured there was only one type of incongruence for each incongruent condition, and no incongruence in the congruent condition. Moreover, the congruent condition was regarded as the baseline for comparison with the other three incongruent conditions (Figure 1).

Tasks were presented using E-Prime presentation software (E-Prime 2.0 Professional, Psychology Software Tools, Inc.) with a predefined pseudo-random stimuli list. Stimuli were presented against a silver background on a screen, viewed at a distance of approximately 60 cm. Participants performed 100 practice trials to ensure they understood the task instructions. The formal experiment consisted of five blocks with 1084 trials in total. Half of the trials were incongruent (i.e., ↑↑↓↑↑ or ↓↓↑↓↓). The trial number ranged from 207 to 221 trials in each block due to variable distances and times of response-rule switching. There were 160 response-rule switching trials: 100 rule-switch trials and 60 non-analyzed switch trials (e.g., the switch trials have other conflicts such as flanker interference or response alternation). The four experimental conditions (i.e., flanker interference, rule-switch, response-alternation, and congruent condition) were assigned pseudo-randomly into each block, with 100 trials for each condition, and all conditions were distributed across the blocks as equally as possible. The trials that were not defined as a conditioning trial might serve as filler trials, which were not analyzed.

A trial started with a cross-fixation of 500 ms, followed by a blank screen for 800–1,200 ms. Then, a stimulus (i.e., a string of five arrows) was presented on the screen until a response or 1,000 ms elapsed. The correct response should be executed within 1,000 ms from the stimulus onset. Finally, a blank screen appeared for 500–800 ms. The total experiment, including electroencephalogram (EEG) preparation, task training, task run, and breaks, took about 2 h.

### Electrophysiological Recording and Analysis

The EEG data were recorded using Brain Amp equipment (Brain Products, Germany) with 64 Ag/AgCl ring electrodes, which were mounted on an elastic cap following the extended 10–20 system. The online reference electrode was placed over FCz, and the ground electrode was placed over AFz. An electrode placed under the right eye allowed the monitoring of blinks and vertical eye movements (VEOG). Electrode impedance was kept below 10 kΩ. Raw data were band-pass filtered between 0.01–100 Hz and digitized at a sampling rate of 500 Hz. The offline preprocessing of the EEG signal was performed in Brain Vision Analyzer 2.1 (Brain Products Gmb H). Data were re-referenced to the average of the two mastoid channels (TP9 and TP10). A semi-automatic Independent Component Analysis (ICA; Vigário, 1997; Shackman et al., 2010) based eye-movement and blink artifact rejection were performed. On average, no more than three ICs were removed from each participant. To reduce high-frequency noise, the signal was low-pass filtered at 30 Hz (slope 2 dB/octave). Next, extracted epochs (from −200 to 800 ms) of the correct trials were time-locked on the stimuli. The resulting data were baseline-corrected using windows from −200 to 0 ms. Furthermore, trials containing further artifacts were removed using an automatic detection criterion for peak-to-peak deflections in a segment exceeding ±80 μV within intervals of ±200 ms. The averaging procedure performed on the extracted epoch. The single-subject ERP averages for each of the four conditions were used for further analysis. The final data set contained at least 62 artifact-free trials per condition for each subject (see more raw waveforms in Supplementary Figure S1).

Based on the relevant literature (Donchin and Coles, 1988; Barceló et al., 2002; Sanders and Lamers, 2002), the stimulus-locked ERPs were measured at three midline electrodes: Fz, Cz, and Pz. Also, the following components were defined: P2/N1 (160–220 ms), N2 (290–370 ms), and P3 (370–600 ms). The visual inspection showed that the P300 latency differed markedly among the conditions. To calculate more accurate P300 amplitudes for each subject in each condition, we detected the P300 latency and analyzed the mean amplitude, which was averaged over a time window of 100 ms symmetrically around the peak of P300 for each subject in each condition (Clayson et al., 2013; Poikonen et al., 2016; Wass et al., 2019). Moreover, to compare the differences between the conditions in terms of response-execution processes after stimulus evaluation, we also analyzed the duration and mean amplitude from P300 peak to keypress. The duration from the P300 peak to keypress was calculated for each condition for each participant by subtracting the P300 latency from the reaction time (RT). All analysis of variances (ANOVAs) were subjected to a 3 (frontality: frontal, central, parietal) × 4 (condition: flanker interference, rule-switch, response-alternation, congruent condition) repeated-measures ANOVA. Greenhouse-Geisser corrections were performed where necessary. All multiple comparisons used Bonferroni correction with a significance level of *p* < 0.05. For brevity, only results related to the conditions were reported.

## Results

### Behavioral Data

The accuracy was calculated for each condition as the percentage of the correct responses of all trials. The mean RT was calculated for the correct responses under each condition. Mean RTs and accuracies for each condition are shown in Table 1. The repeated-measures ANOVA on the RTs and accuracies showed a significant main effect of condition (*F*_RT (2,50)_ = 160.08, *p* < 0.001, $\eta_{\text{p}}^{2}$ = 0.87; *F*_accuracy (1,33)_ = 47.41, *p* < 0.001, $\eta_{\text{p}}^{2}$ = 0.66). Multiple comparisons (Bonferroni correction) indicated that all three types of incongruent conditions yielded longer RTs (all *p* < 0.001) and lower accuracies than the congruent condition (*p*_flanker_ < 0.01, *p*_rule-switch_ < 0.001, *p*_response-alternation_ < 0.05). Moreover, the rule-switch condition yielded longer RTs and lower accuracy than the flanker interference and response-alternation conditions (all *p* < 0.001).

**Table 1:** Mean (standard error) reaction time (RT) and accuracy for each condition.

Condition	Flanker interference	Rule-switch	Response-alternation	Congruent
RT (ms)	597 (10)	684 (11)	577 (10)	516 (9)
Accuracy (%)	95 (0.8)	82 (1.8)	96 (0.4)	98 (0.6)

### ERP Data

**Figure 2:**
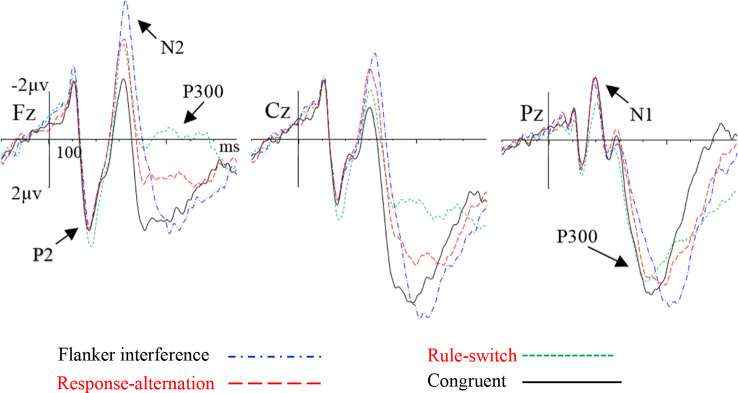
Grand-averaged event-related potential (ERP) waveforms for each condition at the selected electrodes.

**Figure 3:**
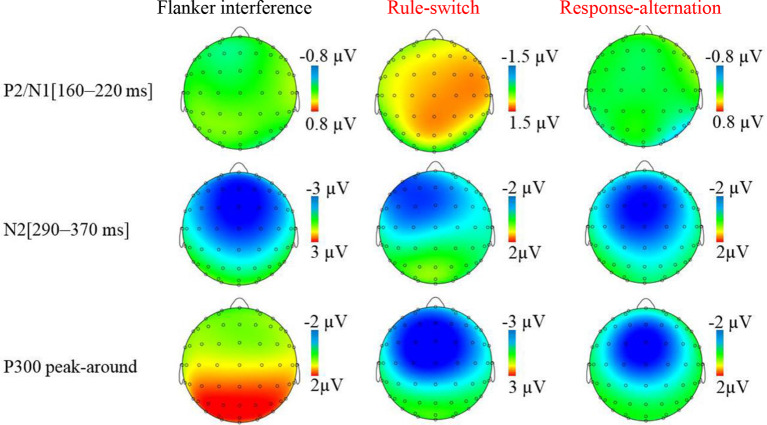
Topographic map of incongruence effect (incongruent condition minus congruent condition) for each incongruent condition in specified time windows.

The grand-averaged ERP waveforms and topographic maps of the incongruence effects (incongruent minus congruent) are shown in Figures 2, 3, respectively. The ANOVA on the mean amplitude in the P2/N1 (160–220 ms) time window showed a significant main effect of condition, *F*_(2,55)_ = 8.56, *p* < 0.001, $\eta_{\text{p}}^{2}$ = 0.26. Pairwise comparisons revealed that the rule-switch evoked larger frontal P2 amplitudes and decreased parietal N1 amplitudes than the congruent (*p* < 0.05), flanker interference (*p* < 0.001), and response-alternation (*p* < 0.01) conditions. There was no significant interaction of condition × frontality.

During the N2 (290–370 ms) time window, ANOVA revealed a significant main effect of condition, *F*_(3,72)_ = 14.29, *p* < 0.001, $\eta_{\text{p}}^{2}$ = 0.37, which was specified by a significant interaction of condition × frontality, *F*_(3,75)_ = 12.24, *p* < 0.001, $\eta_{\text{p}}^{2}$ = 0.34. Further tests showed that, compared to the congruent condition, flanker interference evoked larger N2 amplitudes at the frontal (*p* < 0.001), central (*p* < 0.001), and parietal electrodes (*p* < 0.05); the response-alternation condition evoked larger N2 amplitudes at the frontal (*p* < 0.001), central (*p* < 0.001), and parietal electrodes (*p* < 0.05); the rule-switch condition evoked a larger N2 amplitude at the frontal electrode (*p* < 0.05). Taken together, all three types of incongruence evoked larger N2 amplitudes than the congruent condition in the frontal site. A comparison between the three incongruities revealed that the flanker interference evoked larger N2 amplitudes than the rule-switch at the frontal (*p* < 0.05), central (*p* < 0.01), and parietal electrodes (*p* < 0.01); the response-alternation evoked a larger N2 amplitude than the rule-switch at the parietal electrode (*p* < 0.05). Moreover, flanker interference evoked a larger N2 amplitude than response-alternation at the frontal electrode (*p* < 0.05).

The ANOVA on P300 latency revealed a significant main effect of condition, *F*_(3,72)_ = 189.08, *p* < 0.001, $\eta_{\text{p}}^{2}$ = 0.89. Paired comparisons showed that any two types of incongruence processes differed in P300 latency (all *p* < 0.001). The latency increased gradually from the rule-switch (410 ms) to the response-alternation (451 ms), and flanker interference (526 ms) conditions. The P300 latency in flanker interference and rule-switch differed significantly from that of the congruent condition (all *p* < 0.001), but there was no significant difference in the P300 latency between the response-alternation and the congruent conditions. There was also no significant interaction between condition and frontality.

The ANOVA on the P300 peak-around amplitudes revealed a significant main effect of condition, *F*_(3,72)_ = 28.12, *p* < 0.001, $\eta_{\text{p}}^{2}$ = 0.54, which was specified by a significant interaction of condition × frontality, *F*_(2,54)_ = 18.28, *p* < 0.001, $\eta_{\text{p}}^{2}$ = 0.43. The simple effect analysis revealed that, compared to the congruent condition, the rule-switch condition evoked smaller P300 amplitudes at the frontal and central electrodes (all *p* < 0.001), and the response-alternation evoked smaller P300 amplitudes at the frontal (*p* < 0.001) and central electrodes (*p* < 0.01). A comparison between different types of incongruence revealed that flanker interference evoked larger P300 amplitudes than the rule-switch at the frontal and central electrodes (all *p* < 0.001), and flanker interference evoked larger P300 amplitudes than the response-alternation at the frontal and central electrodes (all *p* < 0.001). Furthermore, the rule-switch condition evoked more attenuated P300 amplitudes than the response-alternation condition at the frontal (*p* < 0.01) and central electrodes (*p* < 0.01).

**Figure 4:**
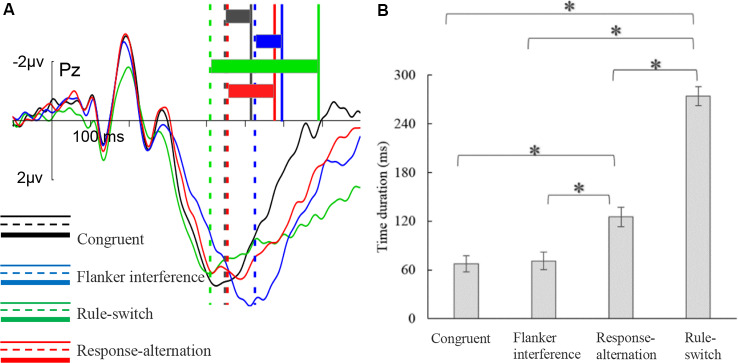
P300peak–keypress time duration for each condition. **(A)** The illustration of the distance from the P300 peak to a keypress. The vertical solid and dashed lines indicate the positions of keypress (RTs) and P300 peak, respectively. The colored rectangles indicate the time duration between P300 peak and keypress in each condition. **(B)** The results of multiple comparisons of P300peak–keypress time duration. The error bars represent standard errors, *p < 0.001.

The ANOVA on the duration from the P300 peak to the keypress revealed a significant main effect of condition, *F*_(2,53)_ = 228.78, *p* < 0.001, $\eta_{\text{p}}^{2}$ = 0.91 (Figure 4). Multiple comparisons showed that flanker interference elicited the shortest duration (71 ms), which was almost the same as under the congruent condition (68 ms), and was significantly shorter than that of the response-alternation (125 ms, *p* < 0.001) condition and the rule-switch (274 ms, *p* < 0.001) condition. Furthermore, the rule-switch condition had a longer duration than the response-alternation (*p* < 0.001) condition. There was no significant interaction between condition and frontality.

Additionally, the ANOVA on the mean amplitude in the P300_peak_–keypress time window showed a significant main effect of condition, *F*_(2,51)_ = 26.87, *p* < 0.001, $\eta_{\text{p}}^{2}$ = 0.53, which was specified by a significant interaction of condition × frontality, *F*_(3,74)_ = 10.60, *p* < 0.001, $\eta_{\text{p}}^{2}$ = 0.31. Further tests showed that, compared to the congruent condition, rule-switch evoked more negative amplitudes at the frontal and central electrodes (both *p* < 0.001), and the response-alternation condition also evoked more negative amplitudes at the frontal (*p* < 0.001) and central electrodes (*p* < 0.01). Between different incongruent conditions, rule-switch evoked more negative amplitudes than the flanker interference condition at the frontal (*p* < 0.001), central (*p* < 0.001), and parietal electrodes (*p* < 0.05); rule-switch evoked more negative amplitudes than response-alternation at the frontal (*p* < 0.01) and central electrodes (*p* < 0.001); response-alternation evoked more negative amplitudes than flanker interference at the frontal (*p* < 0.01) and central electrodes (*p* < 0.001).

## Discussion

In the present study, the ERPs evoked by distinct types of incongruence processes in a hybrid flanker task were compared. The behavioral results indicated that flanker incongruence showed longer RTs and lower accuracy than in the congruent (baseline) condition, which is consistent with the typical findings of the flanker congruency effect (Eriksen, 1995). Compared to the congruent condition, the rule-switch trials showed less accuracy and longer RTs, reflecting the cost of rule switching (Jersild, 1927; Spector and Biederman, 1976; Rogers and Monsell, 1995; Monsell, 2003). The longer RTs and lower accuracy in the response-alternation condition compared to the congruent condition reflect the costs of response alternation (Bertelson, 1963; Meiran, 2000; Schmidt and Liefooghe, 2016). Moreover, the rule-switch condition showed the longest RTs and lowest accuracy among all three types of incongruence, reflecting the difficult and time-consuming processes in rule reversal learning (Schroder et al., 2012; Von der Gablentz et al., 2015; Xie et al., 2017; Shi et al., 2018).

Consistent with our expectation, all three types of incongruence processes evoked larger N2 amplitudes than the congruent condition in the frontal region, implying that a general process—conflict (or incongruence) monitoring—is performed in the frontal brain region (Koechlin et al., 2003; Aron et al., 2007; Hsu et al., 2011). Specifically, when dealing with incongruence, the processes underlying conflict (or incongruence) monitoring and inhibiting of irrelevant information were required, that is, the participants needed to inhibit attention to the distractors from the flankers in the flanker interference condition (Heil et al., 2000; van Veen and Carter, 2002; Clayson and Larson, 2011; Larson et al., 2012; Swick and Chatham, 2014), inhibit the invalid rule in the rule-switch condition (Schroder et al., 2012; Xie et al., 2017), and inhibit the action primed by the preceding trial in the response-alternation condition (Meiran, 2000; Gajewski et al., 2010). Thus, the enhanced frontal N2 in the three types of incongruence supported a general superordinate network in cognitive control (Duncan, 2010; Niendam et al., 2012; Shenhav et al., 2013; Croxson et al., 2009; Vassena et al., 2014; Hampshire and Sharp, 2015; Silvetti et al., 2018). However, the spatial resolution of the electrophysiological method is low, and the spatial distribution of the N2 effects was also different between conditions to some extent. Therefore, the current results only indicate that several different conflicts may induce some general cognitive process, namely conflict monitoring, in the same time window (i.e., N2), but they do not precisely indicate the relationship with the corresponding cortical region.

Although all three types of incongruence evoked a larger N2 component compared to the congruent condition in the frontal region, there were still some exclusive characteristics in each condition. Particularly, the flanker interference condition yielded larger N2 amplitudes than the other types of incongruence processes. van Veen et al. (2001) used event-related fMRI to measure the response of the ACC during a flanker task in which distracting information could be conflicting at the level of stimulus identification or the response level. Although both types of conflict caused a RT interference, the fMRI data showed that the ACC was responsive only to response conflict. These results provide evidence that the ACC, which is an important subregion of mPFC, may respond differently to different types of conflict during conflict monitoring. In the current study, a larger N2 in the flanker interference may reflect the possibility that the conflict monitoring system is sensitive to the explicit conflict information rather than to the implicit conflict that is based on memory updating. Specifically, the incongruence in flanker interference originated from the flanker stimulus, which was more explicit in the current trial. However, the incongruent information in rule-switch and response-alternation were both based on the incongruence of response selection between the preceding and the current trial. Accordingly, the flanker conflict was more explicit (stimulus-based) and the other two were more implicit (memory-based). Also, Verguts and Notebaert (2009) suggested that the enhancement of cognitive control in conflict situations, which represents conflict adaptation, can only transfer to the same S-R binding situation, which was also supported by Feldman et al. (2015). Thus, conflict adaptation is task-specific and cannot be transferred across tasks. In the present study, all three incongruent conditions had a congruent trial N-1, which ensured that cognitive control in all incongruent conditions was not affected by conflict adaptation but by the conflict itself. Therefore, the difference in N2 amplitude between different conditions suggests that the conflict monitoring system can select optimal resources to address specific conflict information (Croxson et al., 2009; Shenhav et al., 2013; Vassena et al., 2014; Silvetti et al., 2018). Additionally, the rule-switch condition elicited smaller N2 amplitudes and the N2 effect was limited in the frontal region, implying that more cognitive resources were allocated to inhibit the invalid rule (i.e., rule reconfiguration, as shown in P300 components), compared to monitoring the conflict information. In rule-switch condition, the fast the abnegation to old response-rule and the update to new response-rule, the better the performance will be. In brief, the cognitive monitoring system might allocate different amounts of cognitive resources when dealing with different types of incongruence, which accounts for the amplitude difference of the conflict-control related N2 component (Lavie et al., 2004; Croxson et al., 2009; Hübner et al., 2010; Shenhav et al., 2013; Vassena et al., 2014; Silvetti et al., 2018; Rey-Mermet et al., 2019).

The distinct neural responses among the three types of incongruence were further confirmed in the P300 time window. First, the P300 latency differed significantly among the different conditions. The flanker interference condition evoked a more prolonged P300 latency than the other types of incongruence, while the rule-switch condition showed the shortest P300 latency. Previous studies have found that the peak latency of P300 is primarily sensitive to the relative duration of the stimulus evaluation process (McCarthy and Donchin, 1981; Magliero et al., 1984; Coles et al., 1985; Smid et al., 1990; Ridderinkhof and van der Molen, 1995). In the present study, the longest P300 latency for the flanker interference trials might have been caused by the considerable time spent evaluating the direction of the target (central) arrow surrounded by a string of distractor arrows. The short P300 latency for the rule-switch trials possibly reflected the facilitated stimulus evaluation caused by the change in the stimulus color, which corresponded to the rule switch. The longer P300 latency for incongruent trials than congruent trials was not generally found in the flanker tasks. The latency results were first found in the most classical and pure flanker task (i.e., letter flanker task), in which the predominant processes were stimulus discrimination and response selection (McCarthy and Donchin, 1981; Smid et al., 1990). The subsequent studies that used the purer flanker task (e.g., arrow flanker task) replicated this result (Doucet and Stelmack, 2000; Van’t Ent, 2002; Hillman et al., 2009; Mückschel et al., 2017). However, there was less focus on or reporting of the relationship between P300 latency and flanker incongruence when investigators adopted the modified flanker task with more complicated stimuli (e.g., emotional words) to study more complicated cognitive processes (Li et al., 2014; Schmitz et al., 2014).

Second, by combining the RTs with the P300 latency, we found that the time length of the P300_peak_–keypress interval in the flanker interference condition was as short as that of the congruent condition. That is, after completion of the stimulus evaluation indexed by P300 latency (Coles et al., 1985), the time used for response preparation and execution in the flanker interference condition was nearly the same as in the congruent condition, implying that in the flanker interference condition the increased RT was not due to the increased time in response-related processing, but due to the increased time in stimulus evaluation. This finding demonstrates that conflict resolution in the flanker interference condition is mainly completed in the stimulus-evaluation stage. In contrast, the time length of the P300_peak_–keypress interval in the rule-switch and response-alternation conditions was longer than that of the congruent condition, suggesting that the increased RTs in these two conditions were due to the increased time in response-related processing.

Third, at the frontal and central sites, the rule-switch and response-alternation conditions elicited a negative-going waveform from the P300 peak to the late ERP complexes as compared with the flanker interference condition. This result might confirm the role of response incongruence in the TSR, for the S-R remapping was similar between the response-alternation and rule-switch conditions (Dreisbach et al., 2002; Koch, 2005). Furthermore, corresponding to the longer P300_peak_–keypress interval for the rule-switch condition relative to the response-alternation condition, the P300 amplitudes were even more attenuated in the rule-switch condition than the response-alternation condition, which might reflect that the degree of S-R remapping was greater in the rule-switch condition than in the response-alternation condition. Specifically, in the rule-switch condition, participants must perform a thorough reconfiguration of the task rule, which not only included a selection of the concrete S-R association (e.g., press “J” for “↓” in the current trial), but also included the establishment of a general rule set that involved activation of the other S-R association (i.e., press “F” for “↑”) that was not used in the current trial. This complex process of reconfiguring the S-R set was more complicated than the response-alternation condition, which only included a concrete S-R remapping.

Finally, electrophysiological differences were also found in the 160–220 ms time window. In this time window, the rule-switch elicited larger fronto-central P2 amplitudes than the other conditions. Previous studies have found that P2 amplitude was sensitive to early rule change and was associated with selective attention to the critical features of stimuli (Rushworth et al., 2002b; Rushworth et al., 2005; Potts, 2004; Astle et al., 2008; Lavric et al., 2008; Lenartowicz et al., 2010; Elchlepp, 2011; Capizzi et al., 2015; Tsai and Wang, 2015). In the present study, the rule-switch condition involved a change in the stimulus feature (i.e., stimulus color) to remind the remapping of response rules. Participants would focus more attention on rule-related perceptual features (i.e., the color of the stimuli), and would have to engage early registration of the need for response remapping (Rushworth et al., 2002b; Lavric et al., 2008). In other words, the early fronto-central P2 amplitudes might reflect the early detection of conflict between the currently-cued task-set and the previous one (Botvinick et al., 1999). Alternatively, the larger frontal P2 amplitude in the rule-switch condition is also functionally similar to the difference positivity component (switch vs. repeat), which is associated with cue-triggered TSR processes (Rushworth et al., 2002b; Karayanidis et al., 2003; Nicholson et al., 2005). Therefore, the P2 possibly reflects the early processes underlying the rule-switch.

In conclusion, a larger frontal N2 amplitude was observed for all three types of incongruence, implying a shared process of cognitive control required for monitoring incongruence or irrelevant information. However, the amplitude and topographical distribution of the N2 effect differed between the different types of incongruence. Also, flanker interference yielded the longest P300 latency, whereas rule-switch and response-alternation yielded smaller P300 amplitudes and shorter P300 latencies. Moreover, the P300_peak_-keypress duration was longer for rule-switch and response-alternation than flanker interference. These findings suggest that various types of incongruence are monitored and resolved by the cognitive control system in similar manners to some extent, although the precise neural dynamics are variable within the specific incongruent information. Moreover, the current study, which was more of an exploratory investigation, adapted a single-factor design to observe the cognitive processes underlying a single piece of incongruent information when compared with that underlying a non-conflict condition. The proportion of multiple-conflicts trials was less than 5.6%, which cannot explore the interaction of different conflicts. In future studies, we intend to use a factorial combination to examine the interactions between different conflicts and to verify the present results.

## Data Availability Statement

All datasets generated for this study are included in the article/Supplementary Material.

## Ethics Statement

The studies involving human participants were reviewed and approved by The Moral & Ethics Committee of the School of Psychology at Jiangxi Normal University (China). The patients/participants provided their written informed consent to participate in this study.

## Author Contributions

LX and FL designed the experiment. ZL and BC conducted the experiment and analyzed the data under the supervision of FL. LX and FL wrote the manuscript. FL edited and revised manuscript. FL approved final version of manuscript. All authors reviewed the manuscript.

## Conflict of Interest

The authors declare that the research was conducted in the absence of any commercial or financial relationships that could be construed as a potential conflict of interest.

## Supplementary Material

FIGURE S1 The raw waveforms for all subjects (*N* = 25) in each condition in Fz electrode.
