# Supplementary figures and images for: Neural Dynamics of Cognitive Control in Various Types of Incongruence
Source: Front Hum Neurosci. 2020 Jun 5;14:214. doi: 10.3389/fnhum.2020.00214 (PMC7291779; doi:10.3389/fnhum.2020.00214)

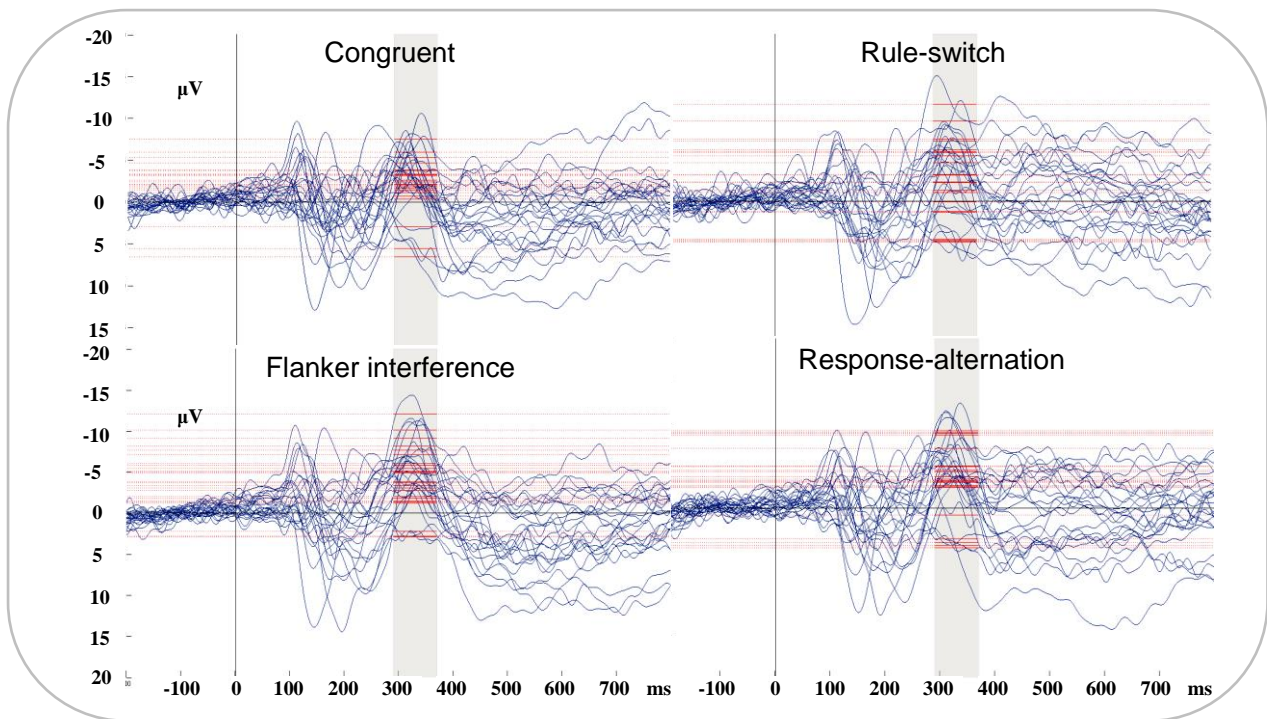

**Figure S1.** The raw waveforms for all subjects ( $N=25$ ) in each condition in Fz electrode.

Supplement: FIGURE S1 — The raw waveforms for all subjects (N = 25) in each condition in Fz electrode. [file Image_1.pdf]
